# Minimally Invasive Colorectal Resection in Kidney Transplant Recipients: Technical Tips, Short- and Long-Term Outcomes

**DOI:** 10.1155/2014/254612

**Published:** 2014-10-28

**Authors:** Sami Alasari, Min Sung Kim, Seung Hyuk Baik, Byung Soh Min, Nam Kyu Kim

**Affiliations:** Section of Colon and Rectal Surgery, Department of Surgery, Yonsei University College of Medicine, 25 Seongsanno, Seodaemun-gu, Seoul 120-752, Republic of Korea

## Abstract

*
Aim*. To prove the safety and feasibility of minimally invasive (laparoscopic and robotic) colorectal resection in kidney recipients by evaluating the technical protocol and reviewing short- and long-term outcomes. *Methods*. Between May 2007 and August 2012, a retrospective review of ten kidney transplant patients diagnosed with colorectal cancer was evaluated for technical tips, short- and long-term outcomes. *Results*. The mean patients' age was 56.8 ± 9.91 years and 50% of them were male. Anterior and low anterior resections were performed in 40% of the patients each; 20% and 10% of the patients underwent right and left hemicolectomy, respectively. Most (90%) procedures were performed laparoscopically and 10% were performed robotically. No open conversions. Mean operating time was 192.5 ± 15 min, blood loss was 30 ± 50 mL, and mean hospital stay was 9.7 ± 5.5 days. Two (20%) patients had postoperative complications: wound seroma and chyloperitoneum. Over a mean follow-up period of 31.4 ± 21.57 months, no mortality or kidney rejection occurred. Among the six patients followed up for a mean of 43.5 ± 9.84 months, 83.3% were 3-year disease-free and the overall survival rate was 100%. *Conclusion*. Minimally invasive colorectal resection is likely to be safe and feasible, with fewer complications and acceptable short- and long-term outcomes, in kidney transplant recipients.

## 1. Introduction

Kidney transplant recipients not only have numerous comorbidities associated with renal failure, but also often have additional problems due to chronic immunosuppression [1–3]. The surgical management of colorectal cancer is risky to the patient in terms of the surgical procedure and the interruption of immunosuppression. In general, these patients are more susceptible to perioperative complications.

Although open colorectal resection has been practiced for many years in kidney recipient patients, the minimally invasive procedure has been developed and is gaining popularity. Multiple randomized controlled trials have proven that minimally invasive surgery yields better short-term outcomes than open colorectal surgery in terms of reduced intraoperative bleeding, postoperative pain, and hospital stay, as well as a lower incidence of infection and respiratory complications, with equivalent long-term outcomes [4, 5]. High-risk patient groups are often excluded from minimally invasive procedures [6]. However, selected high-risk patients may benefit from minimally invasive colorectal resection due to reduced morbidity.

## 2. Aim

The aim of this study was to prove the safety and feasibility of laparoscopic and robotic colorectal resection in kidney transplant recipients by evaluating the technical protocol and the short- and long-term outcomes.

## 3. Methods and Materials

This retrospective review of all kidney transplant recipients diagnosed with colon or rectal cancer who received laparoscopic or robotic resection was conducted between May 2007 and August 2012 at Yonsei University College of Medicine, South Korea. Short- and long-term outcomes were evaluated and technical methods were assessed. Data were analyzed using the SPSS statistical program (version 18 for Windows; SPSS Inc., Chicago, IL, USA). A *P* value ≤ 0.05 was considered statistically significant.

*Technique.* All patients received 4 liters of GoLYTELY (PEG-3350 and Electrolytes for Oral Solution, Braintree Laboratories, Inc., Braintree, MA) preoperatively to evacuate the intestine. Antibiotics and a stress dose of steroids of 100 mg were given on induction of anesthesia and continued postoperatively with tapering dose of steroid. All kidney transplants were performed at the right iliac fossa.

A main concern in laparoscopic and robotic colorectal resection is accurate and safe port placement to avoid injury to the transplanted kidney.

**Figure 1:**
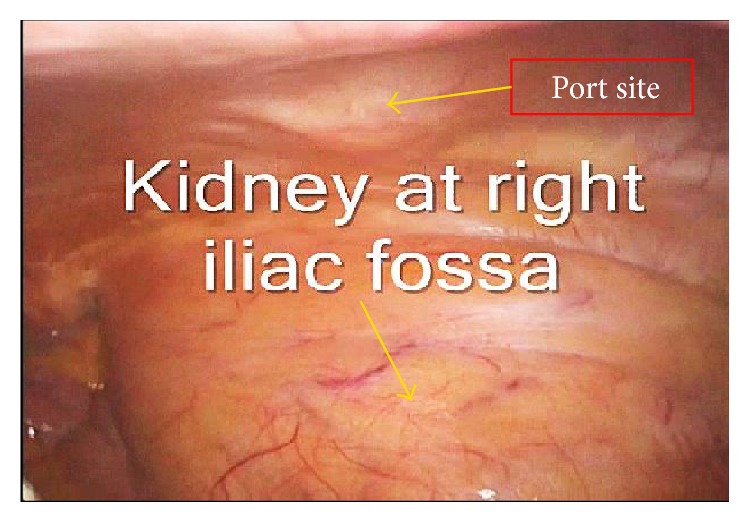
Kidney shifted laterally and posteriorly after CO_2_ insufflation.

After periumbilical port insertion using an open or closed technique, the abdomen was insufflated with medical CO_2_ to 12–14 mmHg pressure. The kidney was shifted posterolaterally to provide adequate space for the working ports (Figure 1). Next, depending on the type of procedure, two right- or left-sided trocars under direct visualization were easily placed. For robotic trocar positioning, we used a hybrid technique by which the colon was mobilized laparoscopically and the robot was docked between the legs of the patients and used mainly for pelvic dissection. Robotic port insertion depended on the location of the kidney: for the right kidney, arms 2 and 3 were on the left and arm 1 was on the right; for the left kidney, arms 1 and 3 were on the right and arm 2 was on the left.

The procedure used colonic mobilization and followed total mesorectal excision principles as required for pelvic dissection. We started medial to lateral mobilization of the colon 2 cm distal to the sacral promontory going upward toward the inferior mesenteric artery where most of it is ligated at its origin. Then, inferior mesenteric vein ligated at the lower border of the pancreas. We carefully identify the ureter and gonadal vessels. Then, lateral dissection followed including splenic flexure mobilization. Finally, we dissect the rectum starting posteriorly going deep to the levator ani muscles carefully to avoid hypogastric nerve injury. Then, we dissect laterally and anteriorly where we should avoid injury to prostrate and neurovascular puddle. Usually, we perform a rectal irrigation before transection. The specimen extracted from the left iliac fossa assistant port and duple stabling technique was used for bowel anastomosis.

Regarding the right colectomy cases, medial to lateral approach our preferred method is applying complete mesocolic principles where vessel ligation is performed at its origin.

## 4. Results

Between May 2007 and August 2012, 782 patients underwent kidney transplantation. Ten of these recipients were diagnosed with colon or rectal cancer and underwent minimally invasive surgical resection. Five (50%) patients were males; the mean patient age was 56.8 (range, 47–72) years, and the mean body mass index was 22.4 (range 20.5–26.4) kg/m^2^. Nine (90%) patients had hypertension, three (30%) had diabetes mellitus, one patient had a history of hepatitis, and one had a history of chronic pulmonary tuberculosis. The main reasons for kidney failure were the hypertension and diabetes mellitus. No patient had a history of liver failure or transplant. The American Society of Anesthesiologists scores were II in 60% of patients and I and III in 20% of patients each.

All rectal patients were evaluated by computed tomography (CT) scan and a colonoscopy. In addition, magnetic resonance imaging (MRI) was used for further evaluation of the patients with rectal cancer. All diagnoses of rectal or colonic adenocarcinoma were made* via* biopsy. Synchronous tumors were identified in one case. Preoperative stages were I and II in 40% of the patients and III in 10% of the patients. No tumor invasion or extension beyond the mesorectal fat with a maximum stage was reported for colon and rectal cancer of T3N1.

Preoperative chemoradiation therapy was administered to one patient with low-rectal cancer with a preoperative stage III (T3N1 M0). Four (40%) patients underwent an anterior and a low anterior resection, two (20%) underwent a covering ileostomy and a right hemicolectomy, and one patient (10%) underwent a left hemicolectomy. Most (90%) procedures were performed laparoscopically and 10% were conducted robotically (Table 1).

Pre- and postoperative laboratory blood findings were evaluated every other day to minimize intra- and postoperative complications (Table 2). The mean carcinoembryonic antigen level was 3.66 ± 2.53 (range 0.72–9.18) ng/mL and the mean white blood cell count was 7, 315 ± 3,842 (range 2,990–16,520) mm^3^. Three (30%) patients were anemic, with a mean hematocrit of 34.65 ± 7.72% (range 26.7–53.4%) and a mean hemoglobin concentration of 11.38 ± 2.54 (range 8.9–17.4) g/dL. Mean preoperative urea and creatinine levels were 27.7 ± 23.62 (range 11.4–89.4) mg/dL and 1.43 ± 0.98 (range 0.59–3.94) mg/dL, respectively.

Most of the immunosuppressive medications used pre- and postoperatively were Cyclosporine or Tacrolimus combined with low dose of steroid ranging between 5 mg to 12 mg. No change in immunosuppressive medication or dosage was documented. The mean postoperative hemoglobin concentration was 10.63 ± 2.45 (range 7.6–16.1) g/dL and no patient required a blood transfusion. The mean postoperative leukocyte count (10,655 ± 2,219 (range 7,530–14,220) mm^3^) was slightly but significantly higher than the preoperative value (*P* = 0.032), and no severe leukocytosis or leukopenia was seen. Mean postoperative urea and creatinine levels were 18.35 ± 17.8 (range 11.4–60.7) mg/dL and 1.40 ± 0.76 (range 0.72–3.09) mg/dL, respectively.

The mean interval between kidney transplantation and surgery was 11.7 (range 1.3–22) years.

Operative data were evaluated. The mean operating time was 192.5 ± 53.87 (range 77–263) min. Operating time was longest (263 min) for the robotic surgery. The mean operating time for laparoscopic surgery was 184.6 ± 50.74 min. The mean estimated blood loss was 30 ± 53.74 (range 0–150) mL. No conversion was required in any of the cases. We rarely found adhesions; most observed were minimal and were noted when the peritoneum was opened accidentally during the kidney transplant or another procedure. Mild adhesions do not contraindicate minimally invasive surgery or indicate an immediate conversion.

No significant difference was observed between pre- and postoperative kidney function parameters, and no organ rejection was reported.

The mean hospital stay was 9.7 ± 5.5 (range 5–25) days. The mean timing of the first oral intake was 3.8 ± 5.81 (range 2–7) days postoperatively. Two (20%) patients had minor postoperative complications: wound seroma and chyloperitoneum. Postoperative stages were I in 20% of the patients, II in 50% of the patients, and III in 30% of the patients (Table 3).

One patient underwent adjuvant chemoradiation (Xeloda (capecitabine) + radiation) and four patients underwent adjuvant chemotherapy alone (fluorouracil, leucovorin, folic acid, oxaliplatin (FOLFOX) in three cases, and 5-fluorouracil + leucovorin in one case).

Over a median follow-up period of 31.4 ± 21.57 (range 2–63) months, no mortality occurred. One patient showed liver metastasis 1 year postoperatively, which was treated by radiofrequency ablation and no further recurrence till the present date of our study. In six patients who were followed for a mean of 43.5 ± 9.84 (range 32–63) months, the 3-year disease-free rate was 83.3% and the 3-year overall survival rate was 100% (Table 3).

## 5. Discussion

The risk of colorectal neoplasia is high among renal transplant recipients and is associated with the duration of immunosuppression, regardless of age [7]. The reported incidence of colorectal cancer in kidney transplant recipients has ranged from 0 to 0.9% [8].

Kim et al. [9] found that the rate of right colon cancer was significantly higher in transplant recipients than in the general cancer population (35.2% versus 15.4%), whereas the rate of rectal cancer was significantly lower in transplant recipients (33.5% versus 46.5%; *P* = 0.031). Kasiske et al. [10] also reported that the risk of colorectal cancer was twofold higher in the first posttransplantation year than in the general population, and an additional 2.2-fold higher after the third posttransplantation year.

In contrast, Saidi et al. [8] found no increased risk of colorectal cancer among transplant recipients compared with the general population and noted the need for further evaluation of this issue.

Parnaby et al. [11] reported that three population-based studies showed increases of up to twofold in the incidence of colonic but not rectal cancer in renal transplant recipients and that two population-based studies showed threefold and 10-fold increases in the incidence of anal cancer in these patients.

Patients on immunosuppressive medications or who have undergone organ transplantation with comorbidities have increased morbidity (stress-related complications) and mortality and a prolonged postoperative recovery period, after major open surgery, including colorectal surgery [12–15].

Wichmann et al. [16] reported a less-pronounced proinflammatory response to surgical trauma after minimally invasive surgery. He concluded that the nonspecific immune response appeared to be less affected by laparoscopic surgery compared with open surgery, while specific cell-mediated immunity was equally affected. Laparoscopic colorectal resection is less physically stressful and offers short-term advantages compared with open resection [17].

Technically, selection of the port insertion site is the most important step in a laparoscopic or robotic procedure. After the establishment of pneumoperitoneum, the kidney is moved inferolaterally to allow port placement. Although unproven, lower pneumoperitoneum pressures seem to help preserve allograft function [18].

Our hospital is one of the leading hospitals in robotic surgery in Korea. As we reported in our results, that one case was performed by robotic method. In that case, we used a hybrid technique where the colonic mobilization was completed laparoscopically and the Da Vinci robot was used only for pelvic dissection. We found that the robotic port position of the 3rd arm should be positioned on the opposite side of the transplanted kidney because of its close proximity with the anterior superior iliac spine where the kidney is located even after pneumoperitoneum. However, any port position even in totally robotic technique could be safe as long as the ports are placed away from transplanted kidney.

We found no or minimal adhesion during the procedures, which may have been related to peritoneum opening during retroperitoneal dissection in the kidney transplant procedure. Adhesions from previous surgeries, possibly related to the use of steroids, were not an issue.

Only one case series of laparoscopic-assisted colectomy in three kidney transplant recipients has been published [19]. In that series, the average time since transplantation was 8 (range 6–10) years and no patient experienced organ rejection. All patients had colon cancer, and all allografts were contralateral to colon resection. The mean operative time was 103 (range 100–105) min and no complications occurred. Renal function remained intact, and termination of immunosuppressive therapy was not necessary. The average hospital stay was 5 (range 4–7) days and the mean follow-up time was 17 (range 3–40) months. Postoperatively, no rejection occurred and the patients remained cancer free.

Our series was larger than that described above, and it included patients with colonic and rectal cancer. Moreover, we included patients who underwent laparoscopic and robotic surgery. In our series, the mean interval between kidney transplantation and diagnosis of colorectal cancer (11.7 years) was slightly longer. Our mean operative time was 192.5 ± 53.87 (range 77–263) min, due to the longer operative time (263 min) of the robotic procedure; the mean operative time for laparoscopic procedures was 184.6 ± 50.74 min. We observed similarly intact renal function and no rejection during the follow-up period. Two patients in our series had minor complications (wound seroma and chyloperitoneum) that were managed conservatively. The leukocyte count is normally raised after any major surgical procedure, but no severe leukocytosis or leukopenia occurred in our patients indicating infection or rejection. No change in the form or dosage of immunosuppressive medications was required before surgery, and medications were resumed postoperatively under monitoring by the transplant team.

In general, these patients are more susceptible to perioperative complications. Immunosuppression can lead to a higher infection rate and surgical stress can increase immunosuppression, a phenomenon that is more pronounced following open (versus laparoscopic) surgical procedures [12, 14, 15].

Our follow-up period (mean 31.4 ± 21.57 months) was longer than that previously reported. In addition, the mean follow-up period was 43.5 ± 9.84 months for the six patients in whom long-term outcomes were assessed. No mortality occurred; the 3-year disease-free rate was 83.3%, and the 3-year overall survival rate was 100%.

In conclusion, minimally invasive surgery is feasible in kidney transplant recipients, who can benefit from fewer wound-related problems. Technical proficiency, experience, and the use of a multidisciplinary team approach including an oncologist and transplant team can result in a successful procedure and ensure the safety of the transplanted kidney. Therefore, minimally invasive colorectal procedures could be considered safe alternatives to open colorectal resection in selected kidney transplant patients.

## Figures and Tables

**Table 1:** Patients' characteristics, type of procedure, and method used.

Variable	
Age (years)	56.8 (47–72)
Sex (male)	5 (50%)
Body mass index (kg/m^2^)	22.4 (20.5–26.4)

	*n* (%)

Comorbidities	
Hypertension	9 (90)
Diabetes mellitus	3 (30)
Hepatitis B	1 (10)
History of old tuberculosis	1 (10)
Liver failure and transplant	0 (0)
Procedure	
Laparoscopic	9 (90)
Robotic	1 (10)
Procedure type	
Anterior resection	4 (40)
Low anterior resection	4 (40)
Right hemicolectomy	2 (20)
Left hemicolectomy	1 (10)

Values are presented as mean (range) or *n* (%).

**Table 2:** Pre- and postoperative laboratory parameters.

Parameter	Preoperative	Postoperative	*P*
	Mean ± SD (range)	Mean ± SD (range)	
CEA (ng/mL)	3.66 ± 2.53 (0.72–9.18)	—	—
WBC (mm^3^)	7,315 ± 3,842 (2,990–16,520)	10,655 ± 2,219 (7,530–14,220)	0.032
Hgb (g/dL)	11.38 ± 2.54 (7.9–17.4)	10.63 ± 2.45 (7.6–16)	0.51
Hct (%)	34.65 ± 7.72 (26.7–53.4)	32.17 ± 8.13 (29.6–24.2)	0.49
BUN (mg/dL)	27.7 ± 23.62 (11.4–89.4)	18.35 ± 17.8 (11.4–60.7)	0.33
Cr (mg/dL)	1.43 ± 0.98 (0.59–3.94)	1.40 ± 0.76 (0.72–3.09)	0.93
Plt (mm^3^)	248,100 ± 88245.17 (119,000–381,000)	214,100 ± 71185.90 (111,000–338,000)	0.35
PT (Sec.)	11 ± 0.88 (9.6–12.3)	11.38 ± 0.97 (9.8–12.5)	0.37
PTT (Sec.)	28.28 ± 3.31 (22.4–34.1)	27.4 ± 2.28 (23.6–30.4)	0.44
INR	0.95 ± 0.083 (0.84–1.09)	0.99 ± 0.089 (0.82–1.11)	0.27

SD: standard deviation; CEA: carcinoembryonic antigen; WBC: white blood cell; Hgb: hemoglobin; Hct: hematocrit; BUN: blood urea nitrogen; Cr: creatinine; Plt: platelets; PT: prothrombin time; PTT: partial thromboplastin time; INR: international normalized ratio.

**Table 3:** Short- and long-term outcomes.

Data	Mean ± SD (range)
OR time (min)	192.5 ± 53.87 (77–263)
EBL (mL)	30 ± 53.74 (0–150)
Conversion	0
LOS (days)	9.7 ± 5.81 (5–25)
1st oral intake day (days)	3.8 ± 1.81 (2–7)

	*n* (%)

Complications	1 (10)
Wound seroma Chyloperitoneum	1 (10)
Postoperative stage	
I	2 (20)
II	5 (50)
III	3 (30)
Recurrence	
Liver metastasis	1 (10)
Mortality	0

3-year DFS (%)	83.3
3-year OS (%)	100

SD: standard deviation; OR: operative; EBL: estimated blood loss; LOS: length of stay; DFS: disease-free survival; OS: overall survival.

## References

[1] Fisher J. S., Woodle E. S., Thistlethwaite J. R. (2002). Kidney transplantation: graft monitoring and immunosuppression. *World Journal of Surgery*.

[2] Chan L., Gaston R., Hariharan S. (2001). Evolution of immunosuppression and continued importance of acute rejection in renal transplantation. *American Journal of Kidney Diseases*.

[3] Hariharan S. (2001). Long-term kidney transplant survival. *The American Journal of Kidney Diseases*.

[4] Lacy A. M., García-Valdecasas J. C., Delgado S. (2002). Laparoscopy-assisted colectomy versus open colectomy for treatment of non-metastatic colon cancer: a randomised trial. *The Lancet*.

[5] The COLOR Study Group (2000). COLOR: a randomized clinical trial comparing laparoscopic and open resection for colon cancer. *Digestive Surgery*.

[6] Schauer P. R., Eubanks W. S., Swanstr L. L., Soper N. J. (2000). Physiologic consequences of laparoscopic surgery. *Mastery of Endoscopic and Laparoscopic Surgery*.

[7] Park J. M., Choi M. G., Kim S. W. (2010). Increased incidence of colorectal malignancies in renal transplant recipients: a case control study. *American Journal of Transplantation*.

[8] Saidi R. F., Dudrick P. S., Goldman M. H. (2003). Colorectal cancer after renal transplantation. *Transplantation Proceedings*.

[9] Kim J. Y., Ju M. K., Kim M. S. (2011). Clinical characteristics and treatment outcomes of colorectal cancer in renal transplant recipients in Korea. *Yonsei Medical Journal*.

[10] Kasiske B. L., Snyder J. J., Gilbertson D. T., Wang C. (2004). Cancer after kidney transplantation in the United States. *The American Journal of Transplantation*.

[11] Parnaby C. N., Barrow E. J., Edirimanne S. B., Parrott N. R., Frizelle F. A., Watson A. J. M. (2012). Colorectal complications of end-stage renal failure and renal transplantation: a review. *Colorectal Disease*.

[12] Allendorf J. D. F., Bessler M., Horvath K. D., Marvin M. R., Laird D. A., Whelan R. L. (1999). Increased tumor establishment and growth after open vs laparoscopic surgery in mice may be related to differences in postoperative T-cell function. *Surgical Endoscopy*.

[13] Bouvy N. D., Marquet R. L., Jeekel J., Bonjer H. J. (1997). Laparoscopic surgery is associated with less tumour growth stimulation than conventional surgery: an experimental study. *British Journal of Surgery*.

[14] da Costa M. L., Redmond H. P., Finnegan N., Flynn M., Bouchier-Hayes D. (1998). Laparotomy and laparoscopy differentially accelerate experimental flank tumour growth. *The British Journal of Surgery*.

[15] Delgado S., Lacy A. M., Filella X. (2001). Acute phase response in laparoscopic and open colectomy in colon cancer: randomized study. *Diseases of the Colon and Rectum*.

[16] Wichmann M. W., Hüttl T. P., Winter H. (2005). Immunological effects of laparoscopic vs open colorectal surgery. A prospective clinical study. *Archives of Surgery*.

[17] Huang C., Huang R., Jiang T., Huang K., Cao J., Qiu Z. (2010). Laparoscopic and open resection for colorectal cancer: an evaluation of cellular immunity. *Gastroenterology*.

[18] Courcoulas A. P., Kelly E., Harbrecht B. G. (1996). Laparoscopic cholecystectomy in the transplant population. *Surgical Endoscopy*.

[19] Rivas H., Martínez J., Delgado S., Lacy A. M. (2004). Laparoscopic assisted colectomies in kidney transplant recipients with colon cancer. *Journal of Laparoendoscopic and Advanced Surgical Techniques*.

